# Derivation and validation of a predictive mortality model of in-hospital patients with *Acinetobacter baumannii* nosocomial infection or colonization

**DOI:** 10.1007/s10096-024-04818-7

**Published:** 2024-04-12

**Authors:** Carola Maria Gagliardo, Davide Noto, Antonina Giammanco, Andrea Catanzaro, Maria Concetta Cimino, Rosalia Lo Presti, Antonino Tuttolomondo, Maurizio Averna, Angelo Baldassare Cefalù

**Affiliations:** 1 Department of Health Promotion, Maternal and Child Health, Internal and Specialized Medicine of Excellence “G. D. Alessandro” (PROMISE), University of Palermo, Via del Vespro 127, Palermo, 90127 Italy; 2 Department of Engineering, University of Palermo, Palermo, Italy; 3 Department of Psychological, Pedagogical, Exercise and Training Sciences, University of Palermo, Palermo, Italy; 4 Institute of Biophysics, National Research Council, Palermo, Italy

**Keywords:** *Acinetobacter baumannii*, Infection, Colonization, Acinetobacter baumannii mortality, Predictive mortality model, Multidrug- resistance

## Abstract

**Purpose:**

*Acinetobacter baumannii* (Ab) is a Gram-negative opportunistic bacterium responsible for nosocomial infections or colonizations. It is considered one of the most alarming pathogens due to its multi-drug resistance and due to its mortality rate, ranging from 34 to 44,5% of hospitalized patients. The aim of the work is to create a predictive mortality model for hospitalized patient with Ab infection or colonization.

**Methods:**

A cohort of 140 sequentially hospitalized patients were randomized into a training cohort (TC) (100 patients) and a validation cohort (VC) (40 patients). Statistical bivariate analysis was performed to identify variables discriminating surviving patients from deceased ones in the TC, considering both admission time (T0) and infection detection time (T1) parameters. A custom logistic regression model was created and compared with models obtained from the “status” variable alone (Ab colonization/infection), SAPS II, and APACHE II scores. ROC curves were built to identify the best cut-off for each model.

**Results:**

Ab infection status, use of penicillin within 90 days prior to ward admission, acidosis, Glasgow Coma Scale, blood pressure, hemoglobin and use of NIV entered the logistic regression model. Our model was confirmed to have a better sensitivity (63%), specificity (85%) and accuracy (80%) than the other models.

**Conclusion:**

Our predictive mortality model demonstrated to be a reliable and feasible model to predict mortality in Ab infected/colonized hospitalized patients.

**Supplementary Information:**

The online version contains supplementary material available at 10.1007/s10096-024-04818-7.

## Introduction

*Acinetobacter baumannii* (Ab) is a Gram-negative opportunistic bacterium responsible for nosocomial infections or colonizations. At present, it is considered one of the most alarming pathogens due to its multi-drug resistance [1]. Ab infections account for ~ 2% of all health care-associated infections in the United States and Europe [2]. Organ localizations of Ab infection involve the respiratory apparatus, with bronchitis and/or pneumonia, the urinary apparatus and the systemic circulation in cases of sepsis. Ab can also grow in various biological fluids, including exudates, skin and soft tissue ulcers, but also in inanimate surfaces (catheters, tracheostomies and/or other devices) due to its biofilm forming properties [3].

The Center for Disease Control and Prevention (CDC) has classified Ab MDR as a serious public health risk, requiring continuous public health monitoring [4]. Moreover, the World Health Organization (WHO) has included carbapenem-resistant Ab (CRAb) in the critical group of bacteria that pose the most serious threat to human health [1]. The mortality rate due to Ab detected in the bloodstream accounts to 44,5% in hospitalized patients, and it increases up to 58% due to CRAb, representing an emerging global health concern [5]. Though CRAb infected patients show a high risk of intra-hospital mortality, CRAb colonized patients present also a high mortality rate (68,5% and 50% mortality rate, respectively for infected and colonized patients) [6]. The current work is aimed to create a predictive model of in-hospital infected or colonized patients by Ab, which could be applied from the first Ab isolation in microbiological samples. The model has been designed to identify patients with higher risk of mortality, deserving prompt antibiotic therapy.

We analyzed Ab alone because of its intrinsic properties: Ab is responsible of sophisticated antibiotic-resistance mechanisms (including carbapenemases and extended-spectrum -lactamases production) and by virulence properties [biofilm forming- activity, host penetration, adherence mechanisms, iron uptake and compartimentalization, presence of polysaccharide membrane and outer membrane protein A (OmpA)] [7]. The last characteristic has been suggested to contribute to host epithelial cells-adhesion, biofilm production, complement resistance [8].

## Patients and methods

### Study design

This study was conducted from 2019 to 2022 and involved two different Units of the Internal Medicine Department of the “Paolo Giaccone” University Hospital of Palermo, Italy.

The first step of the study consisted of the collection of the global sample of patients. The following inclusion criteria were employed: patients aged over 18 years with hospital-acquired Ab infection or colonization. Exclusion Criteria were: patients presenting Ab infection or colonization before ward admission or with positive microbiological cultures to Ab obtained within 48 h from admission; patients with missing admission data, previous Ab targeted antibiotic therapy (90 days from admission).

A total of 140 patients were collected and constituted the global cohort, subjected to a preliminary statistical bivariate analysis. The model was developed in a training cohort (TC) and tested on a separate validation cohort (VC). The predictive power of our custom model was compared with that of the most used predictive scores, as the Simplified Acute Physiological Score (SAPS) II and the Acute Physiological Score Chronic Health Evaluation (APACHE) II.

The patients were randomized into TC and VC with a randomization algorithm created by the “R- version 4.2.2” software. The study then consisted of two phases: the “Training phase” and a second “Validation phase”.

### Data collection and study definitions

The clinical records of patients with microbiological cultures positive for Ab were retrospectively analyzed. Patients’ demographic, clinical, radiological and biochemical data were collected. Clinical data included clinical severity indexes (including SAPS II and APACHE II). Data regarding antibiotic therapy taken in the previous 90 days before admission and during hospitalization, and any other treatment were recorded. Hemodynamic parameters, clinical, biochemical and radiological features were registered both at the time of admission (time zero: ‘T0’) and the time of first Ab isolation from microbiological cultures (time one: ‘T1’).

All patients were screened at admission for colonization by multi-drug resistant (MDR) bacteria by a rectal swab. Microbiological cultures were performed both at admission and during hospitalization in patients with suggestive symptoms for infection, not on a routine basis. Then, colonization/infection was considered only in patients who underwent microbiological cultures. According to the mentioned exclusion criteria, patients with microbiological cultures and/or rectal swabs positive to Ab obtained within 48 h from admission were excluded from recruitment. Isolated Ab were 99% carbapenem-resistant.

An infection was conventionally defined as “pathogen multiplication determining local tissue and organ damage” differing from colonization in which clinical symptoms are not related to the presence of the pathogen itself. The “CDC/NHSN surveillance definition of health care-associated infection and specific types of infections in the acute care setting” criteria were used to discriminate Ab infection or colonization [9]. These criteria differ according to the organ involved and take into account hemodynamic parameters [body temperature, blood pressure (BP), heart rate (HR) and respiratory rate], clinical symptoms and signs (cough, purulent sputum, rhonchi and ranting as regards pneumonia, dysuria, hematuria for urinary infection, pain and signs of local inflammation for skin ulcers), radiological features (X-ray infiltrates, consolidations, abscesses, radiological modification than previous exams), biochemical and microbiological variables [white blood cell count (WBC), positive cultures and relative number of colony-forming units/milliliters- CFU/mL]. Threshold values of CFU/mL differ according to bacterial sources: the threshold is ≥ 10^4^ CFU/mL for bronchoalveolar lavage or protected specimen brushing, ≥10^5^ CFU/mL for urinary culture. Individual organ infection or colonization status was assessed for every single patient. The patient was defined as “colonized” when one or more organs were colonized. The patient was defined as “infected” if at least one infected organ was found, regardless of any other colonized sites. Only the first culture from the same microbiological source was used in the model.

Ab infection or colonization were defined “Hospital-acquired” when Ab was isolated in samples obtained at least 48 h after hospital admission. Patients transferred from an Intensive Care Unit (ICU) ward to our Unit were also included in the study. ICU patients met to the same inclusion/exclusion criteria of internal medicine wards.

The “History of severe organ failure or immunocompromise” category included patients with a NYHA stage IV heart failure, severe chronic lung disease, solid or hematological tumors requiring radiotherapy or chemotherapy, tumor metastases, history of immunosuppression therapy, HIV/IDS, chronic kidney injury requiring dialysis. This definition was borrowed from APACHE II score.

### “Training phase”

**Fig. 1:**
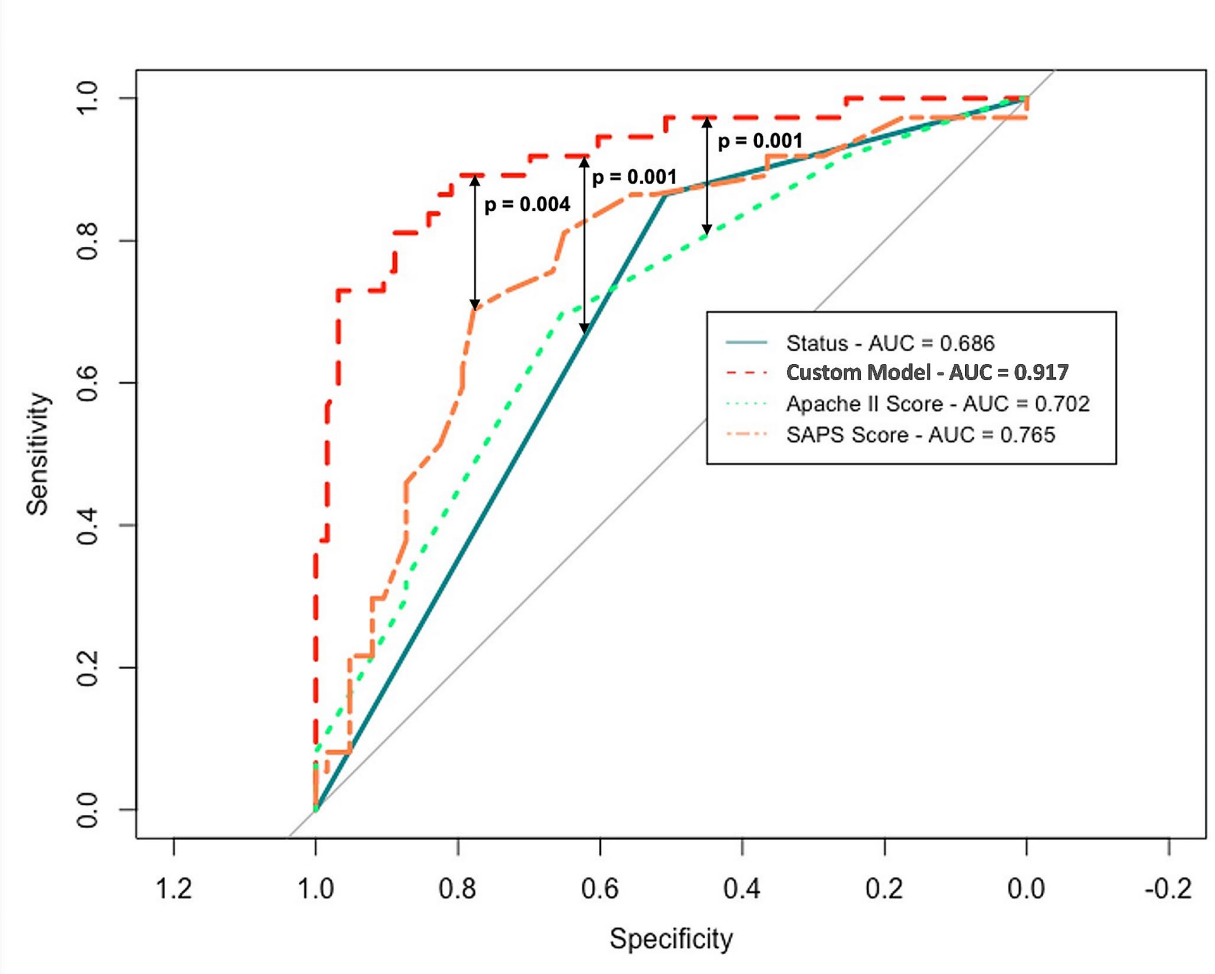
ROC-curves for each predictive mortality model, tested on the “Training cohort”

The cohort of 100 patients was used as TC. The bivariate statistical analysis was performed over this cohort of survivors / deceased patients; the Chi-square test and the two-tailed T-test were applied for categorical and quantitative variables respectively. All analyses were performed with a bilateral alpha risk of 5%. Variables with a bivariate *p* value < 0.1 entered into a logistic regression model as independent variables and were selected using a stepwise backward procedure. “*Exitus”* was the dependent variable of the logistic regression model. The ROC-curves were employed to establish the best cut-off value of the model, according to the Youden Index, to achieve the best compromise between sensitivity and specificity to predict patient survival. APACHE II, SAPS II and the “status model” were chosen as terms of comparison to assess prediction performance. ROC-curves were built for these models too and their best cut-offs were chosen by the Youden index. The “De Long” test was performed to evaluate any statistical difference among the ROC-curves (see Fig. 1). Confusion matrices were also created for each model to better describe how many patients were correctly predicted as “true deceased” or “true survivors” by the models.

### “Validation phase”

The custom model was validated on a second cohort (VC) of 40 patients. The trained model was use in predictive mode in the validation cohort. APACHE II, SAPS II, “Status”, and custom model confusion matrices were also created. The statistical software “R- version 4.2.2” was used for all calculations.

## Results

The overall mortality rate among the 140 patients was estimated as 34.2% (48 out of 140 patient). Among the 140 patients, the “status” (83% in deceased vs. 48% in surviving patients, *p* < 0.001) and “history of severe organ failure or immunocompromise” (69% in deceased vs. 36% in surviving patients, *p* < 0.001) variables resulted significantly different between deceased and surviving patients. BP, HR, peripheral capillary oxygen saturation (SPO2), C-reactive protein (CRP), estimated glomerular filtration rate (eGFR), WBC, neutrophils (N%) and lymphocytes (L%) count, hemoglobin (Hb) and platelets counts (PLT) resulted significantly different at T1 (Table 1).

Also, in the TC (100 patients), the “Status” variable (Ab infection or colonization) resulted significantly different between deceased and surviving patients (T1, *p* < 0.001), with a higher prevalence of infection among deceased patients (Supplementary Table 1). No differences in term of comorbidities, considered both individually and as a burden of comorbidity (Charlson Comorbidity Index), were found in any subgroup. History of severe organ failure or immunocompromise (70% deceased vs. 41% surviving patients, *p* = 0.009) and penicillin antibiotic therapy within 90 days prior to admission (51% deceased vs. 25% surviving patients, *p* = 0.016) were the only categorical variables found to be significantly different at admission in the TC. Urinary leucocyte esterase, nitrites, WBC and PLT resulted significantly different among the quantitative variables at admission (T0). The Glasgow Coma Scale (GCS), PLT, L%, BP, HR, Hb, SPO2, potassium, CRP, GOT transaminases, SAPS II and APACHE II were significantly different between deceased and surviving patients at T1 (Supplementary Table 1).

The different sites of microbiological cultures positive for Ab isolation were tabled according to status (Supplementary Table 2) and patients’ mortality (Supplementary Table 3).

All the significant variables at the bivariate analysis (Table 1) were included in the logistic regression model (Table 2). Status, treatment with penicillin/amoxicillin within 90 days from admission, acidosis, non-invasive ventilation (NIV) (dichotomic variables) and GCS, BP, Hb (quantitative variables) entered the model. The fundamental equation of our model and its variables are illustrated in Table 2.

**Fig. 2:**
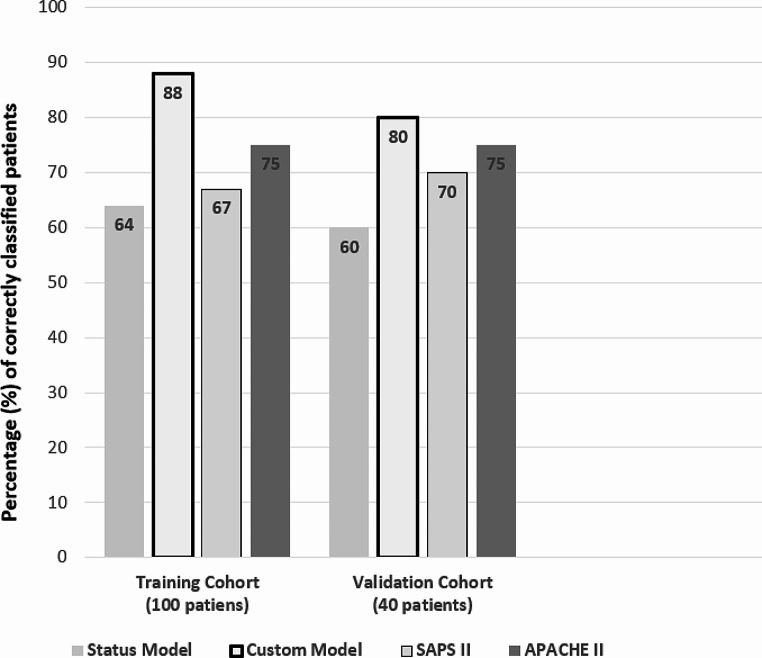
Percentage of correctly classified patients among the models in the TC and VC

ROC curves were built using death or survival as dependent variable. The best cut-off for our model was fixed as 0,66, according to the Youden Index. The “De Long” test identified a statistically significant difference between our model’s ROC-AUC (0.917) and other model’s AUCs (Status: 0.686, APACHE II score 0.702, SAPS II score 0.765) (*p* < 0.001) (Fig. 1). Our model reported a major sensitivity (SE) (93%), specificity (SP) (86%), negative predictive power (NPP), (97%) and accuracy (ACC) (88%) during training, in comparison with the other models (Table 3). It achieved a better performance in comparison with the other models also during the validation phase, in terms of SE (64%), SP (86%) and ACC (80%) (Table 4). The confusion matrices showed the allocation of patients according to the different models. Our custom model performed better in terms of ‘true positives’ (deceased patients) compared to the other models. ( both in the TC of Supplementary Tables 4 and in VC of Supplementary Table 5). Figure 2 summarizes the results of the confusion matrices in a graphical form.

**Table 1:** Differences in entry clinical features between all 140 survived and deceased patients with Ab infection or colonization

Categorical Variables	T0 Deceased *n* = 48	Survivors *n* = 92	p-Value	T1 Deceased *n* = 48	Survivors *n* = 92	p-Value
Sex (Male)	25 (52%)	49 (53%)	1	NA	NA	NA
Age (Years)	76.33 (11.66)	7.17 (11,68)	**0.048**	NA	NA	NA
Pre -hospitalization^A^	20 (41%)	39 (42%)	1	NA	NA	NA
History of severe organ failure or immunocompromise^B^	33 (69%)	33 (36%)	**< 0.001**	NA	NA	NA
Cardiovascular Diseases	41 (85%)	80 (87%)	1	NA	NA	NA
Lung Diseases	21 (44%)	45 (49%)	0.687	NA	NA	NA
Neoplasia	8 (16%)	16 (33%)	1	NA	NA	NA
Liver Diseases	8 (16%)	12 (25%)	0.744	NA	NA	NA
Diabetes	23 (48%)	40 (83%)	0.747	NA	NA	NA
Chronic Kidney Disease	27 (56%)	45 (48%)	0.6	NA	NA	NA
Metabolic Syndrome	23 (48%)	37 (40%)	0.301	NA	NA	NA
90-Days Antibiotics	26 (54%)	52 (56%)	0.931	NA	NA	NA
90-Days Steroids	21 (43%)	32 (35%)	0.393	NA	NA	NA
Immunosuppressive therapy	5 (10%)	5 (5%)	0.459	NA	NA	NA
Bladder Catheter	38 (79%)	69 (75%)	0.733	NA	NA	NA
Tracheostomy	6 (12%)	12 (13%)	1	NA	NA	NA
CVC - PICC – Other devices	13 (27%)	16 (17%)	0.261	NA	NA	NA
NIV use	NEA	NEA	NEA	34 (71%)	32 (35%)	**0.001**
Acidosis	NEA	NEA	NEA	16 (33%)	9 (10%)	**0.001**
Ab Infection	NEA	NEA	NEA	40 (83%)	44 (48%)	**< 0.001**
**Quantitative Variables**	**T0** **Mean (SD)**		***p-Value***	**T1** **Mean (SD)**		***p-Value***
GCS (points)	13.979 (1.604)	14.25 (1.959)	0.382	12.75 (2.564)	14.554 (1.394)	**0.001**
Temperature (C°)	36.558 (0.833)	36.586 (0.807)	0.851	36.742 (0.908)	36.601 (0.816)	0.371
Blood Pressure (mmHg)	118.125 (25.257)	125.659 (26.502)	0.103	110.729 (23.04)	121.75 (22.626)	**0.008**
Heart rate (bpm)	89.417 (17.183)	84.538 (18.67)	0.126	89.125 (15.615)	81.065 (12.37)	**0.003**
SPO2 (%)	93.875 (3.977)	94.076 (4.305)	0.783	93.062 (3.889)	94.859 (2.531)	**0.005**
CRP (mg/L)	92.27 (67.526)	94.971 (90.496)	0.843	117.568 (72.316)	71.036 (63.878)	**0.001**
PCT (mcg/L)	2.179 (6.439)	3.178 (14.75)	0.581	6.705 (17.937)	3.173 (12.808)	0.23
CR (mg/dL)	2.37 (2.452)	2.297 (2.682)	0.872	2.009 (1.595)	1.629 (1.637)	0.189
eGFR (ml/min)	45.006 (31.236)	50.596 (34.698)	0.336	46.917 (32.338)	58.315 (34.66)	**0.056**
Leucocyte urinary Esterase (absolute value)	125.542 (197.005)	156.121 (204.11)	0.392	130.75 (196.717)	108.707 (185.1)	0.522
Nitrites (absolute value)	0.104 (0.371)	0.341 (0.542)	**0.003**	0.188 (1.024)	0.152 (0.49)	0.822
WBC (cells/mcL)	11022.5 (5459.179)	13732.75 (7965.566)	**0.019**	10983.188 (5299.141)	10722.935 (5534.139)	0.786
N (%)	74.713 (17.539)	77.049 (15.103)	0.435	77.267 (19.987)	71.351 (15.313)	**0.077**
L (%)	15.396 (12.043)	14.39 (12.832)	0.648	11.508 (8.279)	18.176 (13,007)	**0.001**
HB (gr/dL)	10.363 (2.286)	10.336 (2.448)	0.949	9.517 (1.688)	10.203 (2.634)	**0.064**
PLT (cells/mcL)	197395.833 (107732.675)	298500.151 (323684.637)	**0.007**	175791.667 (109069.363)	237896.543 (125175.686)	**0.003**
Albumin (gr/dL)	2.875 (0.531)	2.956 (0.563)	0.401	2.872 (0.44)	3.029 (0.513)	**0.061**
Bilirubin (gr/dL)	1.49 (3.335)	0.836 (2.681)	0.244	1.82 (4.96)	0.863 (2.527)	0.215
GOT (IU)	32.021 (28.43)	44.462 (158)	0.468	39.146 (67.082)	22.011 (26.937)	0.095
GPT (IU)	24.604 (25.773)	43.989 (194.582)	0.352	31.292 (45,537)	21.37 (33.418)	0.186
N. Co-pathogens	NEA	NEA	NEA	1.388 (1.139)	1.574 (1.193)	0.382
Charlson Comorbidity Index	7.142 (3.696)	7.972 (2.895)	0.532	NA	NA	NA
APACHE II	29.032 (20.626)	28.351 (19.16)	0.868	28.354 (15.685)	18.478 (10.595)	**0.001**
SAPS II	35.27 (10.401)	40.135 (10.879)	**0.033**	44.625 (9.716)	36.75 (8.377)	**0.001**

The table shows the differences in clinical features, biochemical tests and severity indexes in the initial cohort of 140 deceased and surviving patients with Ab infection or colonization, at the ward admission (T0) and at the time of Ab detection with microbiological cultures (T1). Categorical variables are expressed in term of number and rounded up-percentage %; Chi-square test was applied. Quantitative variables are expressed as Mean and Standard Deviations (SD); Two-tailed T-Test was conducted. NIV: non-invasive ventilation; GCS: Glasgow Coma Scale; SpO2: peripheral capillary oxygen saturation; CRP: C-reactive protein; PCT: procalcitonin; e-GFR: estimated-glomerular filtration rate; WBC: white blood cells; N%: neutrophils; L%: lymphocytes; HB: hemoglobin; GOT, Glutamic-Oxalacetic Transaminase; GPT: glutamic pyruvic transaminase; N. Co-pathogens: number of other bacteria isolated in the same patient, in addition to Ab. APACHE II: Acute Physiological Score Chronic Health Evaluation II; SAPS II: Simplified Acute Physiological Score II. NEA: not evaluated at admission. NA: not available

^A^Pre-hospitalization: hospital discharge by another ward within 90 days from admission in our ward

^B^ History of severe organ failure or immunocompromise: NYHA stage IV heart failure, severe chronic lung disease, solid or hematological tumors requiring radiotherapy or chemotherapy, tumor metastases, history of immunosuppression therapy, HIV/IDS, chronic kidney injury requiring dialysis

**Table 2:** Logistic regression model and variables $L=15.91+{\beta }_{1}\cdot 1.66331+ {\beta }_{2}\cdot 1.37883+ {\beta }_{3}\cdot 1.20416- {\beta }_{4}\cdot 0.84472-{\beta }_{5}\cdot 0.02406- {\beta }_{6}\cdot 0.45139+ {\beta }_{7}\cdot 1.9483$

Variables		Training				
		Coef.		95% C.I.	O.R.	p-value
${\beta }_{1}$	Status (infection or colonization)	1.663		(0.277 ; 3.315)	5.275	0.027
${\beta }_{2}$	Penicillin/amoxicillin within 90 days from admission (YES/NO)	1.378		(0.106 ; 2.763)	3.966	0.038
${\beta }_{3}$	Acidosis (YES/NO) (T1)	1.204		(-0.105 ; 2.592)	3.333	0.075
${\beta }_{4}$	GCS (T1)	-0.844		(-1.468 ; -0.373)	0.429	0.003
${\beta }_{5}$	BP (mmHg) (T1)	-0.024		(-0.053 ; 0.002)	0.976	0.074
${\beta }_{6}$	Hb (gr/dL) (T1)	-0.451		(-0.925 ; -0.034)	0.636	0.044
${\beta }_{7}$	Use of NIV (YES/NO) (T1)	1.948		(0.364 ; 3.77)	7.014	0.022

The table shows the equation the logistic regression model and the entered variables. The dependent variable is the “Exitus” (deceased or survivor). mmHg: millimeters of mercury; gr/dL: grams/deciliters. BP: Blood Pressure. Hb: hemoglobin. NIV: non-invasive ventilation. C.I: confident interval. O.R.: Odds Ratio. T1: Time 1 (Ab isolation time). For numerical variables, ORs are calculated for one unit of measure

**Table 3:** Performance indexes for each predictive morality model tested on the “Training cohort”

Performance Score	“Status” Model	Custom Model	APACHE II	SAPS II
SE	0.508	**0.931**	0.600	0.650
SP	0.865	**0.859**	0.687	0.816
PPP	**0.864**	0.729	0.324	0.702
NPP	0.508	**0.968**	0.873	0.778
ACC	0.640	**0.880**	0.670	0.750
AIC	120	83.750	NA	NA
AUC	0.686	**0.917**	0.702	0.765

Main performance indexes related to the logistic regression models. SE = Sensitivity, SP = Specificity, PPP = Positive Predictive Power, NPP = Negative Predictive Power, ACC = Accuracy, AIC = Model Comparison Performance Index, AUC = Area under the ROC curve. NA: not available

**Table 4:** Performance indexes for each predictive morality model on the “Validation cohort”

Performance Score	“Status” Model	Custom Model	APACHE II	SAPS II
SE	0.380	**0.636**	0.571	0.454
SP	0.842	**0.857**	0.787	0.793
PPP	**0.727**	0.636	0.363	0.454
NPP	0.552	0.857	**0.896**	0.793
ACC	0,6	**0.800**	0.750	0.70

Main performance indices related to the logistic regression models. SE = Sensitivity, SP = Specificity, PPP = Positive Predictive Power, NPP = Negative Predictive Power, ACC = Accuracy, AIC = Model Comparison Performance Index, AUC = Area under the ROC curve

Figure picturing the ROC curve of our tested predictive model, alongside ones of the other predictive models: “Status”, APACHE II and SAPS II indexes. AUC: area under the curve. Statistical differences among the ROC-curves have been evaluated by the “De Long” test.

The graph shows the percentage number of correctly classified patients as deceased or survivors among the models in the TC (100 patients) and VC (40 patients).

## Discussion

The aim of this paper was to create a tool to predict the mortality of hospitalized patients presenting Ab in microbiological samples. The clinical advantage of this predictive tool is to identify the patients at risk that require early an intensive treatment in hospital settings.

In our study sample, mortality rate was 34.2%. This finding is consistent with meta-analyses in literature, in which carbapenem-resistant Ab mortality was estimated as high as 33–44% [5, 10], and a large proportion of the estimated total number of disability-adjusted life-years (DALYs) in Europe Economic Area (EEA) was attributed to colistin-resistant Acinetobacter spp in infected survivors [11].

Polymyxin-class colistin and tigecycline remain the most effective antibiotics to treat CRAb both in ICU and non-ICU wards of Sicilian Hospitals [12]. However, these antibiotic classes are often characterized by several side effects responsible of organ damages. Colistin-induced nephrotoxicity represents a clear example [13]. Recently, Cefiderocol, a siderophore cephalosporin has been used for carbapenem-resistant Gram-negative bacteria. It showed a minor nephrotoxicity compared with colistin, but increased liver enzymes have been observed [14]. To note, the CREDIBLE-CR trial reported higher mortality rate in CRAb patients treated with cefiderocol vs. other therapies (mostly polymyxin based regimens) [15]. So, the identification of hospitalized patients with Ab infection or colonization is a relevant issue that requires a dedicated tool [16]. To our knowledge, no mortality predictive model for Ab have been published yet. Only one predictive score for Ab hospital acquired pneumonia has been developed so far [17].

In the total cohort of 140 subjects, deceased and surviving patients presented similar comorbidities, evaluated both as single diseases and as Charlson Comorbidity Index, as parameter of cumulative burden of comorbidities [18]. Our results suggest that the predictors of mortality are related to clinical characteristics of the patients (Ab infection, use of NIV, previous antibiotic therapy, acidosis, hemodynamic parameters) more than to the degree of inflammation. Probably, concomitant infections by less aggressive bacteria or other inflammatory diseases might explain the increase of inflammation indices without a contextual increase in mortality. Concomitant infections were also investigated as a possible factor influencing mortality, however no influence in mortality was found.

Regarding the variables that entered the model, the presence of penicillin/amoxicillin antibiotic therapy within 90-days before hospitalization suggests that this class of antibiotics might select beta lactamases and carbapenemases producing Ab, responsible for higher mortality rates [18, 19].

“Status” was predictive in our model as well, showing that infected patients have higher mortality rates than colonized ones. However, as mentioned before, high mortality rates were also described in colonized individuals [6], justifying their inclusion in our work.

The variable “acidosis” entered the model, and it has been proven to predict mortality independently from hyperlactatemia [20]. Our data did not discriminate respiratory from mixed or metabolic acidosis. GCS and BP entered the model too, being considered also by different mortality and sepsis score, as the de SOFA and qSOFA scores [21].

Ab biofilm-forming property explained why NIV (versus in-mask O2 supplementation) entered the model as the principal source of infection during hospitalization [22–24].

We compared our custom model with three other models: the “status” alone (infected/colonized), APACHE II and SAPS II. APACHE II and SAPS II have been chosen due to their known association with poor prognosis in Ab infection [18, 25].

Our model correctly classified more deceased/surviving patients (32 out of 40) in comparison with the other models (APACHE II: 30 out of 40, SAPS II: 28 out of 40, “Status” model: 24 out of 40) in the validation cohort (see Supplementary Table 5). Misclassified patients were analyzed: our model incorrectly predicted 8 patients, of whom 4 survived and 4 died. Four out of these 8 patients were equally misclassified by the other models. We could not identify a common clinical pattern that could mislead the model prediction in these patients. The remaining 4 patients (failed by our model) were correctly identified by the other models, and they presented no respiratory tract infections as common characteristic. The absence of lung involvement in these patients may have impaired our model that relies on the variable “NIV” to predict mortality. They also had a high leucocyte count, which was investigated by the other models such as SAPS II and APACHE II and not by our custom model. Nevertheless, inflammatory measures impaired our model performance. In spite of these few cases, our model proved to be globally more performing than competitors.

### Limits of the study

Our sample was very heterogeneous due to the choice of lazily stringent inclusion/exclusion criteria. Still, our sample represents a ‘real world’ experience, depicting the heterogeneity of the patients admitted to ICU and non-ICU hospital wards. We believe that this choice makes the model very exploitable in different hospital settings.

In our ward, patient surveillance was based on rectal swab at admission. It is true that other colonized sites were missed in asymptomatic patient according to this procedure.

Another limit is represented by the size of the cohorts. Larger studies with a higher numerosity of the sample might have refined the result of the present study, producing more accurate parameters estimations able to improve the model performance.

Moreover, the model is applied at T1, that is the moment in which Ab is isolated, before administering antibiotic therapy. The effect of therapy is then not evaluated by the model.

## Conclusions

Our predictive mortality model has been demonstrated to be a reliable and feasible model to identify hospitalized patients with infection or colonization with higher mortality risk at time of detection. The proposed model performs better than other widely used tools, as the infection/colonization status and APACHE II, SAPS II scores. The main strength of our model consists of the use of variables easily available in any hospital setting. If confirmed by further studies, the proposed model might represent a valuable tool to identify patients at high risk, requiring a more aggressive treatment and a higher standard of care.

### Electronic supplementary material

Below is the link to the electronic supplementary material.

Supplementary Material 1

## Data Availability

Not applicable.

## Abbreviations

Ab: Acinetobacter baumannii
CRAb: Carbapenem-resistant Acinetobacter baumannii
TC: Training cohort
VC: Validation cohort
ICU: Intensive Care Unit
qSOFA: Quick SOFA score
SE: Sensitivity
SP: Specificity
AC: Accuracy
AUC: Area under the curve
Spp: Species

## Code availability

The statistical software “R- version 4.2.2” was used.
