# Supplementary material for: Derivation and validation of a predictive mortality model of in-hospital patients with Acinetobacter baumannii nosocomial infection or colonization
Source: Eur J Clin Microbiol Infect Dis. 2024 Apr 12;43(6):1109–18. doi: 10.1007/s10096-024-04818-7 (PMC11178602; doi:10.1007/s10096-024-04818-7)
Supplement: Supplementary file 1 — Supplementary Material 1 [file 10096_2024_4818_MOESM1_ESM.docx]

**SUPPLEMENTARY MATERIAL**

**Derivation and Validation of a** **Predictive Mortality Model of In-hospital patients with *Acinetobacter baumannii* nosocomial infection or colonization.**

**Carola Maria Gagliardo^1^*, Davide Noto^1^*^ǂ^, Antonina Giammanco^1^, Andrea Catanzaro^2^, Maria Concetta Cimino^1^, Rosalia Lo Presti^3^, Antonino Tuttolomondo^1^, Maurizio Averna^1,4^, Angelo Baldassare Cefalù^1^**

1: Department of Health Promotion, Maternal and Child Health, Internal and Specialized Medicine of Excellence "G. D. Alessandro" (PROMISE), University of Palermo, Italy

2: Department of Engineering, University of Palermo, Italy

3: Department of Psychological, Pedagogical, Exercise and Training Sciences, University of Palermo, Italy

4: Institute of Biophysics, National Research Council, Palermo, Italy

*****These authors equally contributed to the present article

**ǂ** corresponding author.

**Corresponding author:** Professor Davide Noto

Address: Department of Health Promotion, Maternal and Child Health, Internal and Specialized Medicine of Excellence "G. D. Alessandro" (PROMISE), University of Palermo. Street: Via del Vespro 127, 90127 Palermo, Italy.

Institution e-mail: [astanteria.mcau@policlinico.pa.it](mailto:astanteria.mcau@policlinico.pa.it)

Tel 0039 091 6554332; Personal e-mail: [davide.noto04@unipa.it](mailto:davide.noto04@unipa.it)

**Supplementary Index:**

1. **Table 1. Differences in entry clinical features between surviving and deceased patients with Ab infection or colonization, at the ward admission (T0) and at the time of Ab detection at microbiological cultures (T1) in the Training Cohort.**
2. **Table 2: Frequencies of A. baumannii isolation sites in the 140 infected and colonized patients**
3. **Table 3: Frequencies of A. baumannii isolation sites in the 140 deceased and surviving patients**
4. **Table 4. Confusion matrixes for each predictive morality model assessed on the “Training cohort”.**
5. **Table 5. Confusion matrixes for each predictive morality model assessed on the “Validation cohort”.**

**Table 1. Differences in entry clinical features between survived and deceased patients with Ab infection or colonization, at the ward admission (T0) and at the time of Ab detection at microbiological cultures (T1) in the Training Cohort.**

|  | T0 | |  | T1 | |  |
| --- | --- | --- | --- | --- | --- | --- |
| Categorical Variables | **Deceased**  **n = 37** | **Survivors**  **n = 63** | ***p-Value*** | **Deceased**  **n = 37** | **Survivors**  **n = 63** | ***p-Value*** |
| Ab Infection | NA | NA | **NA** | 32 (86%) | 31 (49%) | **<0.001** |
| Sex (Male) | 18 (48%) | 32 (51%) | 1 | NA | NA | NA |
| Age (years) | 75.37 (12.57) | 72,84 (11,80) | 0.323 | NA | NA | NA |
| Pre -hospitalization^A^ | 13 (35%) | 24 (38%) | 0.935 | NA | NA | NA |
| History of severe organ failure or immunocompromise^B^ | 26 (70%) | 26 (41%) | **0.009** | NA | NA | NA |
| Normal | 25 (67%) | 47 (74%) | NA | NA | NA | NA |
| Obesity | 9 (24%) | 15 (24%) | NA | NA | NA | NA |
| Cachexia | 3 (8%) | 1 (2%) | NA | NA | NA | NA |
| Cardiovascular Diseases | 31 (84%) | 52 (82%) | 1 | NA | NA | NA |
| Hypertension | 29 (78%) | 49 (77%) | 1 | NA | NA | NA |
| Heart Failure | 12 (%) | 20 (32%) | 1 | NA | NA | NA |
| Ischemic Disease | 9 (24%) | 14 (22%) | 1 | NA | NA | NA |
| Valvulopathy | 4 (11%) | 10 (16%) | 0.685 | NA | NA | NA |
| Arrhythmias | 16 (43%) | 22 (35%) | 0.539 | NA | NA | NA |
| Lung Diseases | 16 (43%) | 30 (48%) | 0.829 | NA | NA | NA |
| COPD | 13 (35%) | 25 (39%) | 0.811 | NA | NA | NA |
| Asthma | 1 (2%) | 3 (5%) | 1 | NA | NA | NA |
| Interstitial Diseases | 1 (2%) | 1 (2%) | 1 | NA | NA | NA |
| OSAS | 1 (2%) | 5 (79%) | 0.53 | NA | NA | NA |
| Neoplasia | 7 (19%) | 12 (19%) | 1 | NA | NA | NA |
| Solid | 4 (11%) | 10 (16%) | 0.685 | NA | NA | NA |
| Hematological | 4 (11%) | 3 (5%) | 0.46 | NA | NA | NA |
| Liver Diseases | 5 (16%) | 11 (17%) | 0.812 | NA | NA | NA |
| Diabetes | 18 (48%) | 28 (44%) | 0.842 | NA | NA | NA |
| Compensated | 9 (24%) | 17 (27%) | NA | NA | NA | NA |
| Not- Compensated | 9 (24%) | 11 (17%) | NA | NA | NA | NA |
| Chronic Kidney Disease | 20 (54%) | 31 (49%) | 0.794 | NA | NA | NA |
| Stage 1 | 1 (2%) | 3 (5%) | NA | NA | NA | NA |
| Stage 2 | 3 (8%) | 4 (6%) | NA | NA | NA | NA |
| Stage 3 | 10 (27%) | 15 (24%) | NA | NA | NA | NA |
| Stage 4 | 1 (2%) | 4 (6%) | NA | NA | NA | NA |
| Stage 5 | 5 (13%) | 4 (6%) | NA | NA | NA | NA |
| Dyslipidemia | 15 (40%) | 19 (30%) | 0.227 | NA | NA | NA |
| Low HDL | 25 (67%) | 43 (68%) | 0.845 | NA | NA | NA |
| Hypertriglyceridemia | 11 (29%) | 11 (17%) | 0.169 | NA | NA | NA |
| Metabolic Syndrome | 19 (51%) | 26 (41%) | 0.273 | NA | NA | NA |
| 90-Days Antibiotics | 20 (54%) | 38 (60%) | 0.687 | NA | NA | NA |
| Oral | 10 (27%) | 22 (35%) | 0.552 | NA | NA | NA |
| Intramuscular | 2 (5%) | 1 (2%) | 0.636 | NA | NA | NA |
| Intravenous | 12 (32%) | 18 (28%) | 0.857 | NA | NA | NA |
| Penicillin | 19 (51%) | 16 (25%) | **0.016** | NA | NA | NA |
| Cephalosporine | 15 (40%) | 24 (38%) | 0.976 | NA | NA | NA |
| Carbapenems | 4 (11%) | 10 (16%) | 0.685 | NA | NA | NA |
| Fluoroquinolones | 5 (13%) | 9 (14%) | 1 | NA | NA | NA |
| 90-Days Steroids | 17 (45%) | 25 (39%) | 0.687 | NA | NA | NA |
| Immunosuppressive therapy | 5 (13%) | 4 (6%) | 0.397 | NA | NA | NA |
| Bladder Catheter | 28 (76%) | 50 (79%) | 0.857 | NA | NA | NA |
| Tracheostomy | 6 (16%) | 8 (12%) | 0.849 | NA | NA | NA |
| CVC - PICC – Other devices | 11 (29%) | 11 (17%) | 0.238 | NA | NA | NA |
| NIV | NEA | NEA | NEA | 27 (73%) | 22 (35%) | **0.001** |
| Acidosis | NEA | NEA | NEA | 14 (34%) | 5 (8%) | **0.001** |
| Quantitative Variables | **T0**  **Mean (SD)** | | ***p-Value*** | **T1**  **Mean (SD)** | | ***p-Value*** |
| GCS | 14.333 (1.704) | 14,081 (1.722) | 0.480 | 14.746 (0.822) | 13 (2,461) | **<0.001** |
| Temperature (C°) | 36.67 (0.896) | 36.627 (0.851) | 0.813 | 36.597 (0.84) | 36,792 (0,961) | 0.309 |
| Blood Pressure (mmHg) | 124.683 (26.653) | 118.243 (24.978) | 0.228 | 121.206 (22.666) | 108.919 (21.671) | **0.009** |
| Heart rate (bpm) | 85.365 (19.307) | 87.919 (17.683) | 0.502 | 80.937 (12,473) | 87.486 (15.68) | **0.034** |
| SPO2 (%) | 94.048 (4.556) | 94.054 (3.972) | 0.994 | 94.778 (2.655) | 93 (4.035) | **0.020** |
| CRP (mg/L) | 91.845 (78.424) | 83.409 (61.341) | 0.552 | 63.355 (56.97) | 120.398 (76.929) | **0.0001** |
| PCT (mcg/L) | 2.479 (12.681) | 1.319 (4.245) | 0.511 | 2.853 (13.362) | 8.176 (20.209) | 0.159 |
| CR (mg/dL) | 2.133 (2.374) | 2.385 (2.534) | 0.625 | 1.586 (1.51) | 2.116 (1.768) | 0.131 |
| EGFR (ml/min) | 53.19 (36.922) | 45.495 (32.756) | 0.283 | 58.952 (36.064) | 48.595 (35.298) | 0.164 |
| Urinary Esterase (absolute value) | 174.258 (202.892) | 75.027 (149.986) | **0.007** | 123.429 (194.826) | 108.811 (177.858) | 0.703 |
| Nitrites (absolute value) | 0.323 (0.505) | 0.081 (0.277) | **0.003** | 0.159 (0.515) | 0.243 (1.164) | 0.678 |
| WBC (cells/mcL) | 14482.794 (8057.41) | 10577.838 (5073.879) | **0.004** | 10483.81 (5824.686) | 11439.541 (5589.87) | 0.419 |
| N (%) | 77.387 (15.446) | 73.162 (18.25) | 0.242 | 69.717 (16.381) | 76.03 (21.53) | 0.129 |
| L (%) | 14.373 (13.4) | 16.27 (12.041) | 0.468 | 20.222 (14.049) | 11.292 (7.457) | **<0.001** |
| M% (%) | 6.919 (4.003) | 9.457 (7.33) | 0.058 | 7.868 (3.186) | 9.276 (12.913) | 0.519 |
| E% (%) | 1.049 (1.517) | 0.978 (1.594) | 0.828 | 1.908 (1.869) | 1.238 (2.157) | 0.120 |
| B% (%) | 0.297 (0.209) | 0.3 (0.297) | 0.961 | 0.438 (1.013) | 0.341 (0.809) | 0.598 |
| HB (gr/dL) | 10.271 (2.442) | 10.278 (2.105) | 0.988 | 10 (1.506) | 9.359 (1.589) | **0.051** |
| Platelets (cells/mcL) | 309396.825 (380610.695) | 186702.703 (106253.331) | **0.019** | 239677.492 (131214.164) | 167891.892 (108323.79) | **0.004** |
| Albumin (gr/dL) | 2.959 (0.604) | 2.902 (0.514) | 0.621 | 3.038 (0.554) | 2.929 (0.458) | 0.291 |
| Na (mmoL/L) | 138.444 (5.233) | 137.459 (6.199) | 0.420 | 140.127 (3.921) | 135.95 (23.122) | 0.283 |
| K (mmoL/L) | 4.161 (1.016) | 4.074 (0.762) | 0.628 | 3.807 (0.706) | 4.19 (0.86) | **0.025** |
| Ca (mmoL/L) | 8.974 (0.836) | 8.847 (1.115) | 0.548 | 8.921 (0.857) | 8.818 (0.84) | 0.560 |
| Bilirubin (mg/dL) | 0.971 (3.239) | 1.635 (3.745) | 0.374 | 1.002 (3.037) | 2.155 (5.616) | 0.254 |
| GOT (IU) | 43.339 (181.002) | 33.892 (31.473) | 0.690 | 18.19 (11.938) | 44.676 (75.548) | **0.041** |
| GPT (IU) | 50.806 (234.481) | 27.514 (28.509) | 0.443 | 19.238 (36.066) | 35.568 (50.998) | 0.092 |
| Charlson Comorbidity Index | 7.38 (3.48) | 7.21 (2.74) | 0.749 | NA | NA | NA |
| N. co-pathogens | NEA | NEA | NEA | 1.377 (1.019) | 1.567 (1.191) | 0.42 |
| APACHE II | 28.576 (20.507) | 28.602 (17.877) | 0.878 | 18.603 (10.197) | 27.892 (14.3) | **0.001** |
| SAPS II | 34.815 (10.4019) | 39.75  (10.157) | **0.008** | 36.762 (8.54) | 45.135 (10.168) | **0.001** |
| Days of hospitalization | NEA | NEA | NEA | 31.05 (22.74) | 20.44 (12.77) | **0.012** |

The table shows the differences in clinical features, biochemical tests, and severity indexes in the Training cohort of 100 deceased and surviving patients with Ab infection or colonization, at the ward admission (T0) and at the time of Ab detection with microbiological cultures (T1). Categorical variables are expressed in term of number and rounded up- percentage %; Chi-square test was applied. Quantitative variables are expressed as Mean and Standard Deviations (SD); Two-tailed T-Test was conducted. NEA: not evaluated at admission; NA: not available; NIV: non-invasive ventilation; GCS: Glasgow Coma Scale; SpO2: peripheral capillary oxygen saturation; CRP: C-reactive protein; PCT: procalcitonin; E-GFR: estimated-glomerular filtration rate; WBC: white blood cells; N%: neutrophils; L%: lymphocytes; M%: monocytes; B%: basophils; E%: eosinophils; HB: hemoglobin; Na: sodium; K: potassium; Ca: calcium; GOT, Glutamic-Oxalacetic Transaminase; GPT: glutamic pyruvic transaminase; APACHE II: Acute Physiological Score Chronic Health Evaluation; SAPS II: Simplified Acute Physiological Score.

^A^Pre-hospitalization: hospital discharge by another ward within 90 days from admission in our ward.
^B^ History of severe organ failure or immunocompromise: NYHA stage IV heart failure, severe chronic lung disease, solid or hematological tumors requiring radiotherapy or chemotherapy, tumor metastases, history of immunosuppression therapy, HIV/IDS, chronic kidney injury requiring dialysis.

**Table 2:** **Frequencies of Ab isolation sites in 140 patients according to infected and colonized status**

| Isolation site | Colonized (n= 56) | Infected (n= 84) |
| --- | --- | --- |
| Blood | **2 (3.5%)** | **40 (47.6%)** |
| Peripheral Vein Blood | NA | 28 |
| CVC | 2 | 12 |
| Lung | **8 (14.3%)** | **22 (26.2%)** |
| Sputum | 4 | 12 |
| BAL/BAS | 4 | 10 |
| Ulcers | **19 (34.0%)** | **20 (23.8%)** |
| Urine (catheter) | **15 (26.8%)** | **30 (35.7%)** |
| Bile | **0%** | **1 (1.2%)** |

The table illustrates the sites of Ab isolation, differentiated according to Ab infection or colonization. For each patient, multiple sites coexist from which Ab is isolated. CVC: Central Venous Catheter; BAL: broncho-alveolar lavage; BAS: broncho-aspirate. NA = positive blood cultures are always considered as infections.

**Table 3:** **Frequencies of Ab isolation sites in 140 patients according to mortality (deceased vs survivors)**

| Isolation site | Survivors (n=92) | Deceased (n=48) |
| --- | --- | --- |
| Blood | **20 (21.7%)** | **24 (50.0%)** |
| Peripheral Vein Blood | 14 | 15 |
| Blood Central Venous Catheter | 6 | 9 |
| Lung | **35 (38.0%)** | **18 (37.5%)** |
| Sputum | 22 | 10 |
| BAL/BAS | 13 | 8 |
| Ulcers | **26 (28.26%)** | **15 (31.2%)** |
| Urine (catheter) | **37 (40.2%)** | **9 (18.8%)** |
| Bile | **0%** | **1 (2.0%)** |

The table illustrates the sites of Ab isolation, differentiated according to Deceased or Survivors patients. For each patient, multiple sites coexist from which Ab is isolated. CVC: Central Venous Catheter; BAL: broncho-alveolar lavage; BAS: broncho-aspirate.

**Table 4. Confusion matrixes for each predictive mortality model assessed on the “Training cohort”.**

| Status | | |
| --- | --- | --- |
| Cut-off = 0.5 | Predicted Value | |
| Real Value | 0 | 1 |
| 0 | 32 | 31 |
| 1 | 5 | 32 |

| Custom Model | | |
| --- | --- | --- |
| Cut-off = 0.66 | Predicted Value | |
| Real Value | 0 | 1 |
| 0 | 61 | 2 |
| 1 | 10 | 27 |

| APACHE II | | |
| --- | --- | --- |
| Cut-off = 0.34 | Predicted Value | |
| Real Value | 0 | 1 |
| 0 | 55 | 8 |
| 1 | 25 | 12 |

| SAPS II | | |
| --- | --- | --- |
| Cut-off = 0.28 | Predicted Value | |
| Real Value | 0 | 1 |
| 0 | 49 | 14 |
| 1 | 11 | 26 |

Confusion matrices (based on the predictions of the Training phase) were built for each model to identify the number of true positives and true negatives, discriminating according to the cut-off, found through the Youden Index of ROC curves. APACHE II: Acute Physiological Score Chronic Health Evaluation; SAPS II: Simplified Acute Physiological Score.

**Table 5. Confusion matrixes for each predictive mortality model assessed on the “Validation cohort.”**

| **Status Model** | | |
| --- | --- | --- |
| Cutoff = 0.5 | Predicted Value | |
| Actual Value | 0 | 1 |
| 0 | 16 | 13 |
| 1 | 3 | 8 |

| **Custom Model** | | |
| --- | --- | --- |
| Cutoff = 0.66 | Predicted Value | |
| Actual Value | 0 | 1 |
| 0 | 25 | 4 |
| 1 | 4 | 7 |

| **SAPS II** | | |
| --- | --- | --- |
| Cutoff = 0.28 | Predicted Value | |
| Actual Value | 0 | 1 |
| 0 | 23 | 6 |
| 1 | 6 | 5 |

| **APACHE II** | | |
| --- | --- | --- |
| Cutoff = 0,34 | Predicted Value | |
| Actual Value | 0 | 1 |
| 0 | 26 | 3 |
| 1 | 7 | 4 |

Confusion matrices (based on the predictions of the Validation phase) were built for each model to identify the number of true positives and true negatives, discriminating according to the cut-off found through the Youden Index of ROC curves. APACHE II: Acute Physiological Score Chronic Health Evaluation; SAPS II: Simplified Acute Physiological Score.
